# Supplementary material for: Responding to COVID-19: The Suitability of Primary Care Infrastructure in 33 Countries
Source: Int J Environ Res Public Health. 2022 Dec 18;19(24):17015. doi: 10.3390/ijerph192417015 (PMC9779330; doi:10.3390/ijerph192417015)
Supplement: Supplementary file 1 [file ijerph-19-17015-s001.zip › ijerph-2085375-supplementary.pdf]

International Journal of
Environmental Research and Public Health

MDPI

Supplementary Materials

Table S1. Safeguarding the well-being of the staff since the COVID-19 pandemic by the practice (n=4380)

	**n**	**%**
Performing triage before patients entering the practice	3259	74,4
Limiting the number of patients in waiting room	3800	86,8
No longer use of the waiting room	539	12,3
Increasing infection control practices	3415	78,0
Structural changes to the reception area	2266	51,7
Performing telephone triage	3475	79,4
Performing video consultations	1702	38,9
Changing repeat prescription approach in terms of patient attending practice	2692	62,5
Using e-script or health mail for prescription	3132	72,7
**Score**		
0	43	1,0
1	131	3,0
2	132	3,0
3	272	6,2
4	507	11,6
5	858	19,6
6	944	21,6
7	943	21,5
8	464	10,6
9	86	2,0
Mean: 5,5; SD: 1,8; Median: 6,0		
